# The Effect of Hyaluronic Acid Gel on Periodontal Parameters, Pro-Inflammatory Cytokines and Biochemical Markers in Periodontitis Patients

**DOI:** 10.3390/gels9040325

**Published:** 2023-04-12

**Authors:** Chenar Anwar Mohammad, Barzan Abdulwahab Mirza, Zainab Salim Mahmood, Faraedon Mostafa Zardawi

**Affiliations:** 1 Periodontics Department, College of Dentistry, Hawler Medical University, Erbil 44001, Iraq; chenar.anwar@hmu.edu.krd (C.A.M.); barzan.abdulwahab@hmu.edu.krd (B.A.M.); zainab.salim@hmu.edu.krd (Z.S.M.); 2 Dean of Faculty of Dentistry, Qaiwan International University, Sulaimani 00964, Iraq; 3 Department of Periodontics, College of Dentistry, University of Sulaimani, Sulaymaniyah 46001, Iraq

**Keywords:** biomedical markers, hyaluronic acid gel, periodontitis, cytokines

## Abstract

Hyaluronic acid in its various forms shows bacteriostatic, fungistatic, anti-inflammatory, anti-edematous, osteoinductive, and pro-angiogenetic properties. This study aimed to evaluate the effect of subgingival delivery of 0.8% hyaluronic acid (HA) gel on clinical periodontal parameters, pro-inflammatory cytokines (IL-1 beta and TNF-alpha) and biochemical markers of inflammation (C-reactive protein (CRP) and alkaline phosphatase (ALP) enzymes) in patients with periodontitis. Seventy-five patients with chronic periodontitis were divided randomly into three groups (25 in each group): group I received scaling and surface root debridement (SRD) + HA gel; group II received SRD + chlorhexidine gel; and group III received surface root debridement alone. Clinical periodontal parameter measurements and blood samples were collected to estimate pro-inflammatory and biochemical parameters at the baseline before therapy and after two months of therapy. The results show that HA gel has a significant effect on the reduction in clinical periodontal parameters (PI, GI, BOP, PPD, and CAL), IL-1 beta, TNF-alpha, CRP, and ALP after 2 months of therapy as compared to the baseline (*p* < 0.05) with nonsignificant differences from the CHX group (*p* > 0.05), except GI (*p* < 0.05), and significant differences from the SRD group (*p* < 0.05). Moreover, significant differences were found between the three groups regarding the mean improvements of GI, BOP, PPD, IL-1β, CRP, and ALP. It can be concluded that HA gel has a positive effect on clinical periodontal parameters and improvements in inflammatory mediators similar to chlorhexidine. Therefore, HA gel can be used as an adjuvant to SRD in the treatment of periodontitis.

## 1. Introduction

Periodontal disease is a general term used to describe specific diseases that affect the gingiva and the supporting connective tissue and alveolar bone, which anchor the teeth in the jaws [1]. The primary etiological factor for periodontal disease is bacterial plaque on the tooth surface that leads to marginal tissue inflammation known as gingivitis. Gingivitis is a reversible condition cured by the mechanical removal of the biofilm and may develop to periodontitis when left untreated [2]. Periodontitis is a multifactorial disease in which putative periodontal pathogens trigger chronic inflammatory and immune responses as a result of the large numbers of bacteria residing in subgingival sulcus in the presence of multiple risk factors [3].

The cytokines interleukin-1beta (IL-1β) and tumor necrosis factor-alpha (TNF-α) are biologically active glycoproteins that are secreted by immune-inflammatory cells in response to inflammation and have a role in the inflammatory process. The release of cytokines through the process of inflammation is considered the most potent factor in periodontal tissue destruction and alveolar bone loss around the involved teeth. The majority of tissue destruction that occurs during periodontitis is attributed to IL-1β and TNF-α activity. Therefore, tissue destruction can be represented as over activity of the host response to periodontal pathogens that stimulates excessive production of IL-1β and TNF-α [4,5]. These cytokines, in addition to interleukin-6 (IL-6), trigger the production of C-reactive protein by hepatocytes [6]. CRP is an acute-phase reactant protein that could be applied as a potential diagnostic tool for gingivitis and periodontitis [7]. A study reported that CRP is an important biochemical marker that has the peculiarity of being involved in periodontal inflammation [7]. ALP is also considered an important biochemical marker that has the peculiarity of being involved in periodontal inflammation [8], which is released from many cells within the area of the periodontium and gingival sulcus and released from polymorphonuclear neutrophils during inflammation, osteoblasts during bone formations, and periodontal ligament fibroblasts during periodontal regeneration.

Surface root debridement is the most effective approach for treating cases of periodontitis [3], which involves removing the biofilm and other bacterial products on the surfaces of the involved roots to minimize the inflammation of gingival tissue and the depth of the periodontal pockets [9]. However, Gontiya and Galgali reported that scaling and root planing (SRP) was technically demanding and was not always efficient in eliminating all periodontal pathogens and in reducing the level of inflammation in periodontitis [10]. This is due to the presence of pathogens within the gingival tissue and in areas anatomically inaccessible to mechanical instrumentation. Therefore, several studies applied systemic and local antimicrobial therapy as an adjunct to mechanical surface root debridement to improve clinical outcomes [11,12,13,14].

The local administration of adjunctive antimicrobial agents to the periodontal pockets showed better clinical improvements, especially in the sites that did not respond to mechanical periodontal therapy alone. A study reported on the effectiveness of local delivery antimicrobial agents (LDAs) in the treatment of periodontitis with the advantages of direct delivery to the site of the infection, reduction in patient complaints, and avoidance of systemic side effects of the antimicrobial agent [15].

One of the most commonly used chemotherapeutic agents against oral diseases is chlorhexidine gluconate. This could be attributed to the wide spectrum of activity of this antimicrobial against all other organisms within the microbial biofilm. However, the prolonged use of chlorhexidine has several side effects, such as teeth staining, disturbances in taste sensation, and increased calculus formation.

The antibacterial and anti-inflammatory activities of hyaluronic acid led to its introduction as a local chemotherapeutic agent with several clinical therapeutic properties for the treatment of periodontitis [10]. A study was conducted to assess the role of free HA molecules in cartilage-bound lubrication through direct examination of the friction between two hydrogenated soy PC (HSPC) lipid layers immersed in a HA solution or two palmitoyl–oleoyl PC (POPC) lipid layers across a HA–POPC solution using a surface force balance (SFB). The results showed that HA addition does not affect the outstanding lubrication provided by the PC lipid layers [16].

Hyaluronic acid, when introduced as a local chemotherapeutic agent, exhibited numerous clinical therapeutic properties [10] and showed anti-inflammatory and antibacterial properties for the treatment of periodontal disease. The use of HA gel after scaling and root planing (SRP) improved clinical periodontitis parameters and colony-forming units (CFUs) compared with the control site treated with only SRP [9,17,18].

HA demonstrates a protective role by limiting the damage that can occur during the inflammatory process. It is acknowledged for its antibacterial, antifungal, and anti-inflammatory effects in addition to its angiogenesis and osteoinductive properties that enhance wound healing in a variety of tissues of the human body, including the periodontal tissues [19]. Further, it enhances tissue regeneration through its water-retention properties in the tissue [20]. The angiogenesis property of HA stimulates bone matrix healing [21]. The high molecular weight of hyaluronan has been shown to stimulate osteoinduction during wound healing. [22]. Several studies investigated the effect of hyaluronan on gingival tissue healing in patients with periodontitis using gingival and periodontal parameter measurements [23,24].

The effect of HA on the serum level of pro-inflammatory cytokines and host response to inflammation biomarkers had not been previously investigated; therefore, the objective of this study was to evaluate the effect of subgingival administration of 0.8% hyaluronic acid gel (GENGIGEL^®^) as an adjunct to surface root debridement on clinical periodontal parameters, pro-inflammatory cytokines (IL-1 beta and TNF-alpha), and biochemical disease markers (CRP and ALP) in a group of patients with periodontitis in comparison with CHX and SRD groups.

## 2. Results and Discussion

The effect of hyaluronic acid on periodontal parameters had been reported by several studies [19,23,24] but without evaluation of its effect on pro-inflammatory cytokines and biochemical markers of inflammation. Therefore, the current study was conducted to evaluate the effect of subgingival administration of 0.8% hyaluronic acid gel as an adjunct to SRP on periodontal parameters, pro-inflammatory cytokines, and biochemical parameters in patients with periodontitis. Our study showed that HA gel had a significant effect on the reduction of clinical periodontal parameters, pro-inflammatory cytokines, and inflammatory disease markers in comparison with chlorhexidine, and significant differences were found between the three studied groups.

### 2.1. Participant Characteristics

Seventy-five patients (44 males and 31 females; 37.3 ± 8.1 years) were divided randomly into three groups: HA test group (14 males and 11 females; 37.4 ± 7.3 years), CHX positive control group (15 males and 10 females; 36.4 ± 7.8 years), and SRP negative control group (15 males and 10 females; 38.2 ± 9.6 years).

### 2.2. Clinical Periodontal Parameters

Table 1 shows that the mean values of the clinical periodontal parameters were significantly decreased after 2 months of therapy in the HA group as compared to those of the base line before therapy. These values significantly decreased from 1.74 ± 0.31 to 0.84 ± 0.21 in PI (*p* = 0.001), from 1.40 ± 0.24 to 0.92 ± 0.17 in GI (*p* = 0.001), from 51.60 ± 24.72% to 11.00 ± 5.07% in BOP (*p* = 0.001), from 4.16 ± 0.21 mm to 3.20 ± 0.25 mm in PPD (*p* < 0.001), and from 4.74 ± 1.05 mm to 3.74 ± 0.85 mm in CAL (*p* < 0.001), as presented in Table 1. The same pattern can be observed in the CHX group after CHX gel application and in the SRD group, where there was a significant decrease in the mean values of all the mentioned clinical parameters.

### 2.3. Mean Differences in Clinical Periodontal Parameters

Table 2 shows the mean differences between the readings taken before therapy and after therapy. The largest mean difference in PI was 0.90 in the CHX group followed by 0.84 in the HA group, and the least was 0.82 in the SRD group with no significant differences between each two compared groups or between the three groups (*p* = 0.528). Regarding GI, the largest improvement was in the CHX group (the mean decrease was 0.78) followed by the SRD group (0.58), and the least improvement was in the HA group (0.48) with significant differences between the HA and CHX groups and between the three groups (*p* = 0.027). Regarding BOP, the largest mean improvement was in the CHX group (58%) followed by the HA group (40%) and SRD group (36%) with non-significant differences between the HA and CHX groups and significant differences between the three groups (*p* = 0.004). For PPD, the largest improvement was detected in the CHX group (decrease of 1.04) followed by the HA group (0.96 mm) and SRD group (0.90 mm) with non-significant differences between the HA and CHX groups; in addition, the difference between the three groups was significant (*p* = 0.020). For CAL, the mean difference was 1.1 mm in the CHX group followed by 1.00 mm in the HA and SRD groups with no significant differences detected between each two compared groups or between the three studied groups (*p* = 0.374).

The significant improvement of gingival (GI and BOP) and periodontal (PPD and CAL) parameters in the HA group may have been due to the anti-inflammatory effects of the HA gel. The subgingival administration of the HA gel resulted in a reduction in prostaglandins, matrix metalloproteinases, and bioactive materials that led to a lessening of tissue destruction and promotion of periodontal tissue healing [25]. The present findings are similar to the results obtained by Al Shammari et al. [26], who reported that the mean values of clinical parameters (GI, PPD, and CAL) were improved in both test groups (scaling and root planing (SRP + 0.8% HA gel)) and control group (SRP alone) but with more improvement in the SRP + HA group [26]. Moreover, a study that investigated the effects of HA on periodontal parameters in patients with chronic periodontitis reported significant improvements of BOP and PPD indices in the HA group as compared with the non-HA group [9]. Another study reported significant improvement of clinical gingival (gingival bleeding index (GBI) and gingival inflammation) and periodontal (pocket depth and clinical attachment gain) parameters after subgingival application of 0.8% HA gel in addition to root debridement due to the bacteriostatic effect on certain pathogens [23]. Furthermore, a study reported that the local delivery of hyaluronan gel in conjunction with scaling and root planing may have a beneficial effect on periodontal health in patients with chronic periodontitis. [27]. In contrast, a study reported that HA gel had no positive effect on periodontal health [28].

The current study also showed that there was a significant reduction in all clinical periodontal parameters after two months of CHX gel application. Moreover, there was no significant difference between the CHX and HA groups in regard to the mean improvements of all clinical periodontal parameters with the exception of the more significant improvement of GI in the CHX group (*p* = 0.026); this may have been due to the fact that, since CHX has a significant broad spectrum of antimicrobial effects, it reduced the number of both facultative and obligate anaerobes in the plaque. These changes in the subgingival microbiota are effective in reducing intraoral plaque and improving the clinical parameters that are associated with gingivitis (GI and BOP) and periodontitis (PPD and CAL) [29]. A study reported on the bactericidal effect of CHX gel, stannous fluoride gel, and amine fluoride gel containing 1.25% fluoride on the subgingival microflora in 40 periodontal pockets of 10 patients. The results showed that the total microbial count was reduced in all groups but with more reduction found in the pockets treated with CHX gel or stannous fluoride gel than in the pockets treated with a placebo gel. The study concluded that a reduction of more than 99% in the microflora of periodontal pockets occurred within 30 min after 2% CHX gel or 4% stannous fluoride gel application [30].

Also, the present study showed that all clinical periodontal parameters were significantly reduced after therapy with SRD alone with no statistically significant differences from the HA group. This may have been due to the positive effect of scaling and root planing (SRP) on decreasing the pathogens present in the periodontal pocket and altering the microflora to become less pathogenic. These results are similar to those from other studies [31,32]. The current results agree with a study that reported the local application of HA and placebo gels as adjuvant to subgingival instrumentation resulted in statistically significant clinical and microbiological improvements as compared to baseline with better results in the HA group and no statistically significant differences between the two groups [33]. Another study found that SRP had a positive significant effect on the reduction in gingival inflammation and healing of periodontal tissue after 4 weeks of therapy in patients with chronic periodontitis and reported that SRP may disrupt subgingival biofilm, allowing the microbial population to shift towards being more associated with health [34]. A study revealed that regular scaling and root planing combined with the patients’ daily home care procedures can resolve gingival inflammation and treat most mild forms of periodontitis [35]. Other studies reported that the successful long-term slowing of periodontal disease progression after treatment depends primarily on regular scaling and root planing by the dentist and on the daily plaque removal by the patient [36,37]. Lindhe and Nyman revealed that, when optimal mechanical removal of bacteria was achieved, the disease did not recur after treatment [36].

### 2.4. Pro-Inflammatory Cytokines and Biochemical Parameters

Table 3 shows that, after HA gel application, the mean levels of IL-1β and TNF-α were significantly decreased from 199.70 pg/mL to 59.16 pg/mL and from 19.68 pg/mL to 18.09 pg/mL, respectively (*p* = 0.001). Regarding biochemical parameters, the mean levels of CRP and ALP were significantly decreased from 4.66 U/L to 3.03 U/L and from 77.00 mg/L to 71.60 mg/L, respectively (*p* = 0.001; *p* = 0.010). The same pattern could be observed in the CHX group, where the mean levels of all mentioned parameters were significantly decreased after 2 months of CHX gel application. In the SRD group, there was also significant reduction in the mean levels of IL- 1β (*p* = 0.007), TNF-α (*p* = 0.001), and CRP (*p* = 0.023) after 2 months of SRD therapy with the exception of the non-significant reduction in the ALP level (*p* = 0.122) (Table 3).

### 2.5. Mean Differences in Pro-Inflammatory Cytokines and Biochemical Parameters

Table 4 shows that the mean difference in the IL-1β level was largest after local application of hyaluronic acid gel in the HA group (40.54 pg/mL) followed by CHX gel application in the CHX group (23.50 pg/mL) with no significant differences between the two groups (0.436). The least decrease in the IL-1β level was detected in the SRD group (12.62 pg/mL), the difference from the hyaluronic acid group was significant (*p* = 0.034), and the difference between the three studied groups was significant (*p* = 0.040). Regarding the TNF-α level, the mean difference was highest in the CHX group (5.80 pg/mL) followed by the HA group (2.64 pg/mL) with no significant differences between the two groups. The least mean reduction in the TNF-α level was detected in the SRD group (1.59 pg/mL) with non-significant differences between the three studied groups (*p* = 0.074). Regarding CRP, the highest mean reduction in CRP was detected in the CHX group (2.89 U/L) followed by the HA group (1.63 U/L) with non-significant differences between the two groups (*p* = 0.283). These decreases were significantly higher than the decrease in CRP in the SRD group (0.30). The differences between the CHX and HA gel groups and SRD group were significant (*p* < 0.001, *p* = 0.015, respectively). Nearly the same pattern was observed in ALP with the mean reduction being largest in the CHX group (12.00 mg/L) followed by the HA group (5.40) with non-significant differences between the two groups (*p* = 0.108). The least mean reduction in ALP was detected in the SRD group (2.20 mg/L) with non-significant differences from the HA group. However, the difference between the three studied groups was significant (*p* = 0.001 (Table 4).

The significant reductions in the mean levels of pro-inflammatory cytokines (IL-1β and TNF-α) and biochemical markers (CRP and ALP) after HA gel application in the HA group with no significant differences from the CHX group may have been due to the significant anti-inflammatory effects of HA, since hyaluronic acid gel has a role in reducing and moderating the inflammatory response through its interaction with the hyaladherin TNF-stimulated gene-6 (TSG-6). TSG-6 is stimulated by the inflammatory cytokines IL-1 beta and TNF-alpha, causing fibroblasts and other inflammatory cells to express the TSG-6 protein [38]. Once expressed by cells, TSG-6 proteins are retained in HA-rich environments and bind to high-molecular-weight HA polymers that form heavy chains. These heavy HA chains prevent inflammation by inhibiting neutrophil migration and plasmin through negative feedback loops [38]. In addition, the anti-inflammatory effect of HA may have been due to the action of exogenous hyaluronan as a scavenger through draining prostaglandins, metalloproteinases, and other bioactive molecules [39]. The anti-inflammatory effect of hyaluronic acid was also investigated, and it was confirmed that the use of HA decreased the levels of cytokines and oxidative stress and resulted in reductions in TNF-alpha and IL-1 beta levels [40].

A study reported that the commercially available high-molecular-weight HA products are highly biocompatible and, in gingival tissues, do not impair the healing process by prolonging gingival inflammation or causing excessive matrix mettalloproteinase expression at the repaired site [41].

As a consequence of the reduction in pro-inflammatory cytokine levels, serum levels of CRP were also reduced. Also, serum levels of ALP were reduced after HA gel application, and this may have been due to the reduction in the numbers of PMNLs brought about by the reduction in gingival inflammation.

Since cells in the periodontal tissue, such as monocytes/macrophages and fibroblasts, have the capacity to produce prostaglandins in response to lipopolysaccharide (LPS) challenge as well as IL-1 beta and TNF-alpha after treatment with chlorhexidine, the level of PGE2 in GCF diminishes due to the decreased effect of lipopolysaccharide and the inflammatory mediators IL-1 beta and TNF-alpha [42]. Also, the finding of the least mean difference in terms of reduction in pro-inflammatory cytokines (IL-1 beta and TNF-alpha) and biochemical disease markers (CRP and ALP) in the SRD group indicated that scaling and surface root debridement alone was not as effective in reducing inflammatory mediators as the other two therapies.

## 3. Conclusions

The present results indicate that the subgingival administration of HA gel as adjuvant to SRD has significant anti-inflammatory effects with a potential role for the treatment of chronic periodontitis through its significant positive impact on clinical periodontal parameters, pro-inflammatory cytokines, and biochemical marker improvement as compared to chlorhexidine. Therefore, the local delivery of HA gel into the periodontal pocket can be recommended as an adjunct to SRD for the treatment of patients with periodontitis, for patients with a known allergy to CHX, and for patients in the periodontal maintenance phase.

## 4. Materials and Methods

### 4.1. Materials (HA and CHX Gels) Composition

Gengigel (Ricerfarma) contains high-molecular-weight HA that is present as sodium hyaluronan (molecular weight: 1.03 ± 0.01 g/cm^3^ at 20 °C), which was considered as the test gel. CHX gel consists of 900 ppm F- and 0.2% chlorhexidine digluconate and the ingredients aqua, hydroxyl ethyl cellulose, Laureth-23, chlorhexidine digluconate, sodium fluoride, aroma, and sodium saccharin.

### 4.2. Study Design and Subject Groups

This clinical comparative study was conducted on 75 systemically healthy participants with chronic periodontitis of both sexes (44 males and 31 females) with ages ranging from 30–60 years, attending the Periodontic Department of the College of Dentistry-Hawler Medical University from February 2021 to July 2021. The study was approved by the institutional ethical committee of the College of Dentistry-Hawler Medical University, Erbil, Iraq.

**Figure 1:**
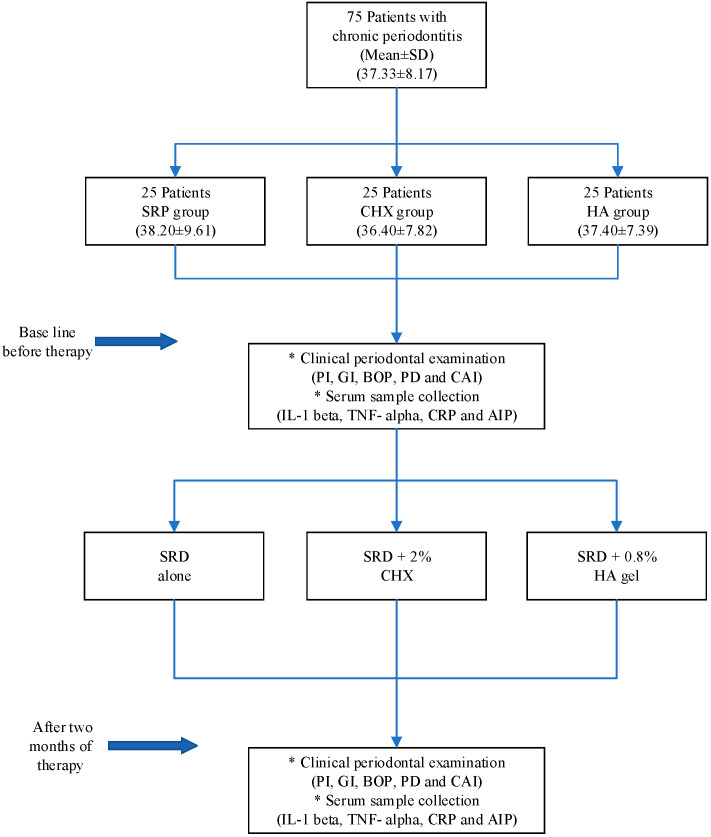
Flowchart of the study protocol. * Indicate the two main tasks of the research, the clinical periodontal parameters measurments and serum sample collection.

The volunteers were divided randomly into three groups: group I (test group) included 25 patients who received subgingival administration of 0.8% hyaluronan gel (GENGIGEL^®^ Ricerfarma, Milano, Italy) after surface root debridement (SRD) and after 1 week post-therapy; group II (positive control group) included 25 patients who received subgingival administration of 0.2% CHX gel (Cervitec gel, Ivoclar Vivadent, Liechtenstein) after SRD and after 1 week of post-therapy; and group III (negative control group) included 25 patients who received surface root debridement alone, called the SRD group. All patients received debridement of root surfaces using Woodpecker dental ultrasonic Piezo scalers (UDS-J) and Nordent hand scalers and Gracey curettes. Figure 1 presents a flowchart of the study protocol showing the selected patients, groups, treatments, and two-month follow-ups of the patients.

In the HA group, the tested sites were softly dried by using an air syringe and isolated with cotton rolls followed by subgingival application of 0.8% HA into the selected sites after scaling and root planing. The 0.8% HA gel was delivered in preloaded 1 mL syringes with a blunt cannula (Gengigel^®^ 0.8%, Ricerfarma). The CHX application consisted of 0.2% CHX gel, which was administered locally into the periodontal pockets by syringe with a 24-gauge needle attached to it.

The gel was inserted gently into the periodontal pockets until the pocket sites were filled. Then, the pocket opening was covered with a periodontal dressing (Coe-Pak^®^) for 1 week to retain the gel in the pocket as well as to prevent the ingress of oral fluids. After Coe-Pak^®^ application, each subject was instructed to report immediately if the pack became dislodged before the scheduled recall visit or if any sort of discomfort, pain, burning sensation, or allergic reaction occurred. After the 1st week recall, the periodontal dressing was removed, and the gel was reinserted into selected pocket sites and covered pby Coe-Pak^®^ for another week with reinforcing of oral hygiene maintenance instructions. Recall visits were conducted at the end of 2 weeks for Coe-Pak^®^ removal.

The inclusion criteria were patients who were systemically healthy, had at least 20 teeth with a probing pocket depth of ≥4 mm and clinical attachment loss ≥ 2 mm in at least 40% of the analyzed sites [43], were free from dental caries, exhibited no known allergies, had not received orthodontic treatment, and had the ability to attend follow-up visits at regular intervals. Pregnant or lactating women, smokers, alcoholics, patients with chronic systemic disease (diabetes mellitus, hypertensive, rheumatoid arthritis), and patients who had received antibiotics or periodontal therapy during the last 6 months were excluded from the study. Data and personal information related to the medical and dental histories of the subjects were obtained from the patients’ responses to the questionnaire. Informed written consent was obtained from each participant before conduction of the study.

### 4.3. Periodontal Parameters Assessment

The examination of the clinical periodontal parameters was carried out for all participants by a single specialized examiner at base line before therapy and after 2 months of therapy. The clinical periodontal parameters included plaque index (PI), gingival index (GI), bleeding on probing (BOP), probing pocket depth (PPD), and clinical attachment loss (CAL). Probing pocket depth was assessed by gentle insertion of the periodontal probe from the gingival margin to the base of the pocket, and CAL was assessed by measuring the distance from the cemento–enamel junction (CEJ) to the base of the pocket by the periodontal probe [44]. The thickness of plaque was measured according to the PI [45] and given a score from 0–3. The extent and severity of gingival inflammation was measured according to the GI [46] through inspection by naked eyes and by gentle probing for six gingival surfaces of the examined tooth and given a score from 0–3, while BOP was performed by running a periodontal probe gently along the inner surface wall of the gingival sulcus and noting bleeding after 30 s as absent (0) or present (1) [47].

The periodontal parameters were recorded at six sites for each tooth with the exception of the third molar, three for buccal (disto-buccal/labial, mesio-buccal ⁄labial, mid-buccal/labial), and three for lingual (disto-lingual/palatal, mesio-lingual/palatal, mid-lingual/palatal) by using a manual periodontal probe (PC-PUNC 15 Hu-Friday, Chicago, IL, USA) for GI, BOP, PPD, and CAL assessments and a straight explorer probe (DenMat, California - United State of America (USA) for PI measurements. The clinical examination was conducted in a dental chair under standard conditions of light using a disposable mouth mirror and dental probe.

### 4.4. Blood Sample Collection

A blood sample (5 mL) was collected from each participant through a disposable syringe and transferred into a gel tube. Then, the sample was centrifuged at 3000 rpm for 15 min to obtain a clear supernatant. The clear supernatant was aspirated and transferred into 4 separate test tubes to measure TNF-alpha (pg/mL), IL-1 beta (pg/mL), CRP (mg/L), and ALP (U/L) at base line before therapy and after 2 months of therapy. The standard methods for the assays (CRP and ALP) were based on the guideline provided by the reagent manufacturer (Human Gm Bh, (Wiesbaden - Germany) of HumaStar 300, a fully automated analyzer, whereas serum levels of IL-1 beta and TNF-alpha were assessed with commercially available enzyme-linked immunosorbent assay kits (ELISA kits) according to the manufacturer’s instructions (Elabscience, Houston, TX, USA).

### 4.5. Statistical Analysis

Data were analyzed using SPSS (Statistical Package for Social Sciences; SPSS Inc., IBM, Armonk, NY, USA) version 26.0. Normality of the data was checked using the Shapiro–Wilk test; accordingly, non-parametric tests were used when indicated. The Wilcoxon signed-rank test was used to compare the means of the same sample but at two different time periods. The Kruskal–Wallis test was used to compare the mean rankings of the three groups, and a post-hoc test (Dunn–Bonferroni) was used to compare each two groups. A *p* value of ≤ 0.05 was considered statistically significant.

## Figures and Tables

**Table 1:** Clinical periodontal parameters before and after therapy for the three studied groups.

		Before Therapy		After Therapy		
Groups	Index	Mean ± SD	Median	Mean ± SD	Median	*p* *
HA	PI	1.68 ± 0.38	1.50	0.84 ± 0.21	1.00	0.001
	GI	1.40 ± 0.24	1.40	0.92 ± 0.17	1.00	0.001
	BOP (%)	51.60 ± 24.72	60.00	11.00 ± 5.07	10.00	0.001
	PPD (mm)	4.16 ± 0.21	4.00	3.20 ± 0.25	3.00	<0.001
	CAL (mm)	4.74 ± 1.05	5.00	3.74 ± 0.85	4.00	<0.001
CHX	PI	1.74 ± 0.31	1.80	0.84 ± 0.21	1.00	0.001
	GI	1.84 ± 0.36	1.80	1.06 ± 0.12	1.00	0.001
	BOP (%)	71.00 ± 14.78	75.00	13.00 ± 6.21	10.00	0.001
	PPD (mm)	4.30 ± 0.62	4.00	3.26 ± 0.54	3.00	<0.001
	CAL (mm)	4.70 ± 0.77	4.50	3.60 ± 0.60	3.50	<0.001
SRD	PI	1.76 ± 0.40	1.80	0.94 ± 0.12	1.00	0.001
	GI	1.58 ± 0.29	1.50	1.00 ± 0.00	1.00	0.001
	BOP (%)	53.60 ± 26.79	50.00	17.20 ± 17.11	10.00	0.001
	PPD (mm)	4.04 ± 0.08	4.00	3.14 ± 0.29	3.00	<0.001
	CAL (mm)	4.10 ± 0.69	4.00	3.10 ± 0.69	3.00	<0.001

HA: Hyaluronic acid; CHX: Chlorhexidine; SRD: Surface root debridement; PI: Plaque index; GI: Gingival index; BOP: Bleeding on probing; PPD: Probing pocket depth; CAL: Clinical attachment loss; *: Wilcoxon signed-rank test; *p* ≤ 0.05 means statistically significant.

**Table 2:** Mean differences (before and after therapy) in clinical parameters for the three studied groups.

Indices	Groups	Mean Difference ^††^	±SD	*p* *	Groups	*p* **
PI	(A) HA	0.84	0.25		A vs. B	0.367
	(B) CHX	0.90	0.35	0.528	A vs. C	0.367
	(C) SRD	0.82	0.37		B vs. C	0.713
	Total	0.85	0.32			
GI	(A) HA	0.48	0.29		A vs. B	0.026
	(B) CHX	0.78	0.25	0.027	A vs. C	1.000
	(C) SRD	0.58	0.29		B vs. C	0.218
	Total	0.61	0.30			
BOP (%)	(A) HA	40.60	21.40		A vs. B	0.054
	(B) CHX	58.00	11.62	0.004	A vs. C	1.000
	(C) SRD	36.40	18.89		B vs. C	0.004
	Total	45.00	19.79			
PPD (mm)	(A) HA	0.96	0.08	0.020	A vs. B	0.059
	(B) CHX	1.04	0.08		A vs. C	1.000
	(C) SRD	0.90	0.21		B vs. C	0.036
	Total	0.97	0.15			
CAL (mm)	(A) HA	1.00	0.33	0.374	A vs. B	1.000
	(B) CHX	1.10	0.21		A vs. C	0.367
	(C) SRD	1.00	0.00		B vs. C	0.461
	Total	1.03	0.22			

PI: Plaque index; GI: Gingival index; BOP: Bleeding on probing; PPD: Probing pocket depth; CAL: Clinical attachment loss; HA: Hyaluronic acid; CHX: Chlorhexidine; SRD: Surface root debridement; *: Kruskal–Wallis test; **: Post-hoc test (Dunn–Bonferroni test); ^††^: The mean ranks were considered in the Kruskal–Wallis test; *p* ≤ 0.05 means statistically significant.

**Table 3:** Immunological and biochemical parameters before and after therapy for the HA, CHX, and SRD groups.

Groups	Before Therapy			After Therapy			
Parameters	Mean	±SD	Median	Mean	±SD	Median	*p* *
HA							
IL-1β (pg/mL)	199.70	81.73	170.80	159.16	44.63	142.40	0.001
TNF-α (pg/mL)	19.68	1.44	19.60	18.09	0.92	17.80	0.001
CRP (mg/L)	4.66	0.98	4.20	3.03	0.83	2.90	0.001
ALP (U/L)	77.00	8.98	73.00	71.60	7.87	71.00	0.010
CHX							
IL-1β (pg/mL)	180.98	24.44	193.00	157.48	22.68	163.50	0.001
TNF-α (pg/mL)	23.72	8.15	21.40	17.92	0.86	17.30	0.001
CRP (mg/L)	7.09	3.19	6.40	4.20	2.14	6.00	0.010
ALP (U/L)	89.60	8.80	93.00	77.60	10.83	82.00	0.001
SRD							
IL-1β (pg/mL)	159.16	21.20	163.00	146.54	14.93	143.90	0.007
TNF-α (pg/mL)	21.44	0.72	21.40	18.80	0.85	19.00	0.001
CRP (mg/L)	5.14	0.80	4.90	4.84	1.16	4.90	0.023
ALP (U/L)	66.20	15.40	61.00	64.00	10.82	60.00	0.122

HA: Hyaluronic acid; CHX: Chlorhexidine; SRD: Surface root debridement; IL-1β: Interleukin-1 beta; TNF-α: Tumor necrosis factor- alpha; CRP: C-Reactive protein; ALP: Alkaline phosphatase enzyme; *: Wilcoxon signed-rank test; *p* ≤ 0.05 means statistically significant.

**Table 4:** The mean differences in the biochemical parameters (before and after therapy).

Indices	Groups	Mean Difference ^††^	±SD	*p* *	Groups	*p* **
IL-1β (pg/mL)	(A) CHX	23.50	7.73		A vs. B	0.436
	(B) HA	40.54	38.47	0.040	A vs. C	0.846
	(C) SRD	12.62	15.66		B vs. C	0.034
	Total	25.55	26.51			
TNF-α (pg/mL)	(A) CHX	5.80	7.44		A vs. B	0.074
	(B) HA	2.64	0.96	0.074	A vs. C	0.595
	(C) SRD	1.59	1.54		B vs. C	0.041
	Total	3.34	4.68			
CRP (mg/L)	(A) CHX	2.89	2.87		A vs. B	0.283
	(B) HA	1.63	1.31	<0.001	A vs. C	<0.001
	(C) SRP	0.30	0.45		B vs. C	0.015
	Total	1.61	2.09			
ALP (U/L)	(A) CHX	12.00	3.14		A vs. B	0.108
	(B) HA	5.40	6.30	0.001	A vs. C	0.001
	(C) SRD	2.20	5.05		B vs. C	0.382
	Total	6.53	6.39			

HA: Hyaluronic acid; CHX: Chlorhexidine; SRD: Surface root debridement; IL-1β: Interleukin-1 beta; TNF-α: Tumor necrosis factor-alpha; CRP: C-Reactive protein; ALP: Alkaline phosphatase enzyme; *: Kruskal–Wallis test; **: Post-hoc test (Dunn–Bonferroni test); ^††^: The mean ranks were considered in the Kruskal–Wallis test.

## Data Availability

All the raw data are available with the authors on a rescannable demand.
